# Congenital absence of the vas deferens with hypospadias or without hypospadias: Phenotypic findings and genetic considerations

**DOI:** 10.3389/fgene.2022.1035468

**Published:** 2022-11-09

**Authors:** Jianzheng Fang, Xiaoyi Wang, Xueping Sun, Yugui Cui, Feiyang Diao, Xiaoyu Yang

**Affiliations:** ^1^ State Key Laboratory of Reproductive Medicine, The Center for Clinical Reproductive Medicine, The First Affiliated Hospital of Nanjing Medical University, Nanjing, China; ^2^ Core Facility Center of the Affiliated Hospital of Nanjing Medical University, Nanjing, China

**Keywords:** congenital absence of the vas deferens (CAVD), *CFTR*, hypospadias, whole-exome sequencing (WES), mutation

## Abstract

Congenital absence of the vas deferens (CAVD) is a major cause of obstructive azoospermia. Mutations of *CFTR* and *ADGRG2* cause the majority of CAVD. Despite this, 10%–20% of CAVD patients remain without a clear genetic diagnosis. Herein, the *CFTR* and *ADGRG2* genes were first sequenced using Sanger sequencing in 50 CAVD patients. Whole-exome sequencing (WES) was used to further identify potential novel genetic causes in CAVD with hypospadias. In total, 29 of 50 CAVD patients carried at least one *CFTR* mutation, but no *ADGRG2* mutation was found. 5T was found to be the most frequent variant in our CAVD populations. Seven CAVD patients with hypospadias were further analyzed using WES. No homozygous or compound heterozygous mutations related to disorders of sex development (DSDs) or male infertility were identified by WES. CAVD with hypospadias presented lower testicular volume (9.71 ± 2.14 ml vs. 14.45 ± 2.93 ml, *p* < 0.001) and higher FSH level (FSH: 7.28 ± 3.91 IU/L vs. 4.24 ± 1.96 IU/L, *p* = 0.027) than CAVD without hypospadias. It is worth noting that neither *CFTR* or *ADGRG2* mutation nor homozygous or compound heterozygous gene mutations were identified in seven CAVD cases with hypospadias. However, nine heterozygous or hemizygous mutations were selected as potential pathogenic genes in CAVD with hypospadias. In conclusion, *CFTR* variants, especially 5T, play a major role in the Chinese CAVD population*.* CAVD with hypospadias shows relatively lower testicular spermatogenesis, suggesting a different genetic basis or pathogenic factor from cystic fibrosis/CAVD or unilateral renal agenesis/CAVD.

## Introduction

Congenital absence of the vas deferens (CAVD) is found in 1%–2% of infertile men (Jequier et al., 1985). The clinical features of CAVD mainly present congenital bilateral absence of the vas deferens (CBAVD) or congenital unilateral absence of the vas deferens (CUAVD), the partial or total absence of the epididymal corpus and cauda, and absence or agenesis of seminal vesicles in the reproductive system (Weiske et al., 2000; Bieth et al., 2021). CAVD is generally identified during the evaluation of infertility due to azoospermia, known as one of the manifestations in cystic fibrosis (CF) (CF/CAVD) or accompanied by unilateral renal agenesis (URA) (URA/CAVD).

CF is a frequent disease in Euro-descendant populations, occurring in 1 of 2,000 newborns (de Souza et al., 2018). In Asian populations, the incidence varies greatly from 1:10,000 to 1:40,750 newborns among different countries (Powers et al., 1996; Yamashiro et al., 1997). Guo et al. reported that no more than 100 CF patients of Chinese origin were reported in the literature, which shows the incidence varies among different ethnic groups (Guo et al., 2018). A meta-analysis showed that the URA frequency in CUAVD patients was 26.8% compared with 6.7% in CBAVD patients (Cai et al., 2019).

The cystic fibrosis transmembrane conductance regulator (CFTR) was first identified by positional cloning in 1989, and its mutation was subsequently associated with CF and CAVD (Riordan et al., 1989; Dumur et al., 1990; Anguiano et al., 1992). Previous research demonstrated that *CFTR* homozygous or compound heterozygous variants (including the 5T allele) were as high as 78% in CBAVD patients, while only 46% in CUAVD patients (Yu et al., 2012; Cai et al., 2019). Almost all CF-related CBAVD patients had *CFTR* homozygous or compound heterozygous variants. X-linked *ADGRG2* gene mutation has been verified to be associated with CAVD in recent years and is responsible for approximately 20% of CAVD not related to *CFTR* mutation (Patat et al., 2016; Bieth et al., 2021). However, *CFTR* or ADGRG2 or other mutations are rarely identified in CAVD men with URA, suggesting that other phenotypes with CAVD might have a different genetic background and need to be further researched.

Hypospadias is another common congenital malformation presented with hypoplasia of the penis. Compared with healthy children, boys born with hypospadias more often have other congenital anomalies. Hypospadias with CAVD is rarely reported in the literature. To our knowledge, hypospadias combined with the absence of the epididymis and vas deferens is only presented in complete androgen-insensitivity syndrome (CAIS) caused by androgen receptor mutation (Gottlieb et al., 1993). However, compared to CAVD patients, CAIS usually has impaired spermatogenesis. Hence, we assume that hypospadias with CAVD might have a genetic basis or pathogenic factor different from that of CF/CAVD and URA/CAVD.

The aim of this study was to evaluate the clinical features of the CAVD population, explore the mutation spectrum in CAVD patients, and further find potential causative genes by whole-exome sequencing (WES) in CAVD with hypospadias.

## Materials and methods

### Patients

A total of 50 patients with CAVD were involved in this research, from January 2018 to December 2021 in the reproductive medicine center, the First Affiliated Hospital of Nanjing Medical University. All patients underwent a complete physical examination by the same physician. Semen volume, pH, and azoospermia were evaluated using semen analysis. The sex hormone levels and karyotyping analysis were obtained by patients. Ultrasound examination was performed to evaluate the upper urinary tract, especially the kidney, and the external genitalia, including the testes, epididymis, and vas deferens. Transrectal ultrasonography was used to assess the seminal vesicles, ejaculatory ducts, and prostate. The diagnosis of CAVD was based on the following criteria: impalpable scrotal vas by physical examination and rectal/scrotal ultrasound showing absence of the vas and/or seminal vesicle. We classified the patients into hypospadias CAVD and non-hypospadias CAVD groups. The testicular volume, follicle-stimulating hormone (FSH), testosterone, and the proportion of CFTR mutation were compared between the two groups. The study was approved by the ethics committee of the First Affiliated Hospital of Nanjing Medical University.

### Pre-screening for cystic fibrosis transmembrane conductance regulator and *ADGRG2* for congenital absence of the vas deferens using Sanger sequencing

Genomic DNA was extracted from peripheral blood lymphocytes using TIANGEN RelaxGene Blood DNA (TIANGEN, Beijing, China) according to the manufacturer’s protocol. *CFTR* and *ADGRG*2 gene mutation analysis was performed by sequencing the coding exons and the exon–intron boundaries of the genes. DNA was amplified by PCR using specific primers for the *CFTR* and *ADGRG*2 genes. PCR was performed using an ABI 9700 PCR system, and the conditions were one cycle of 94°C for 5 min, the first 10 cycles of denaturing at 94°C for 40 s, annealing at 65°C for 30 s, and elongation at 72°C for 1 min, then 25 cycles of denaturing at 94°C for 40 s, annealing at 60°C for 30 s, elongation at 72°C for 40 s, and a final cycle of 72°C for 10 min. The PCR products were sequenced by the ABI 3100 Avant device (Applied Biosystems). The sequencing data were analyzed by comparing the sequences with those reported in Gene Bank NM_000492 (*CFTR*) and NM_001079858.3 (*ADGRG2*).

### Whole-exome sequencing and genetic analysis

WES samples were prepared using IDT xGen Exome Research Panel V1.0 (Integrated DNA Technologies). The Qubit 2.0 fluorometer (Thermo Fisher Scientific) was used to assess the number of sequencing libraries. The 2100 Bioanalyzer High Sensitivity DNA assay (Agilent Technologies) measured the size and quality of libraries. Eligible libraries were loaded into the Illumina NovaSeq platform (Illumina, San Diego, United States). FASTQ files and the human genome reference (hg19) were aligned by BWA v0.7.13. Genotyping of single nucleotide variants and indels from recalibrated BAM files was performed using GATK 4.0 (McKenna et al., 2010) and annotated with ANNOVAR (Wang et al., 2010) in multiple databases, consisting of population frequencies, HGVS variant descriptions, phenotypes or diseases, and variant function predictions. Candidate pathogenic variants were filtered according to the following criteria: 1) exonic and splicing variants; 2) variants with minor allele frequency of less than 0.01 in the human population genome datasets (e.g., the gnomAD and 1000 Genomes Project); 3) variants with good sequencing quality (genotype quality value >150 generated from GATK 4.0 (McKenna et al., 2010) and variant allele frequency >0.2); 4) non-synonymous variants; 5) variants with high expression in human testes; and 6) missense variants predicted to be deleterious by SIFT, PolyPhen-2, and MutationTaster.

### Statistical analysis

Data were analyzed and expressed as mean ± SD using SPSS version 18.0 (IBM Corp., United States). The independent *t*-test was used to compare the means between the two groups. Statistical significance was considered as *p* < 0.05.

## Results

A total of 50 patients with CAVD were involved in this research, including 46 patients with CBAVD and four patients with CUAVD. Seven out of 50 CAVD patients had a history of hypospadias repair. Except for one patient who had chronic sinusitis, the other CAVD patients had no CF-related clinical features, such as progressive obstructive lung disease with bronchiectasis, pancreatic insufficiency, and others.

### Clinical features of congenital absence of the vas deferens patients with hypospadias or without hypospadias

In subgroup analysis, clinical features of hypospadias or non-hypospadias CAVD groups were compared. In the hypospadias/CAVD group, the mean age at the time of assessment was 27.83 ± 3.87 years, ranging from 24 to 35 years. In the non-hypospadias/CAVD group, the mean age at the time of assessment was 29.65 ± 4.27 years, ranging from 24 to 44 years. In the hypospadias/CAVD group, two of seven had a unilateral absence of the vas deferens, while there was only one case of 36 in the non-hypospadias group. In both groups, most of the cases verified by ultrasonography had only caput remnant and agenesis or absence in seminal vesicles. The semen analysis demonstrated azoospermia and lower pH and volume in all the patients. The mean testicular volume assessed by ultrasound was lower in the hypospadias/CAVD group than in the non-hypospadias/CAVD group (9.71 ± 2.14 vs. 14.45 ± 2.93 ml, *p* < 0.001). In the evaluation of hormones, the hypospadias/CAVD group had higher FSH levels and lower testosterone than in the non-hypospadias/CAVD group (FSH: 7.28 ± 3.91 vs. 4.24 ± 1.96 IU/L, *p* = 0.027; T: 10.38 ± 2.45 vs. 12.92 ± 4.29 nmol/L, *p* = 0.192) (Table 1).

**TABLE 1:** Comparison between hypospadias CAVD and non-hypospadias CAVD.

	Hypospadias CAVD	Non-hypospadias CAVD	P
Patients (n)	7	43	—
Age (yr)	27.83 ± 3.87	29.65 ± 4.27	0.22
Testicular volume (ml)	9.71 ± 2.14	14.45 ± 2.93	0.001
FSH (IU/L)	7.28 ± 3.91	4.24 ± 1.96	0.027
T (nmol/l)	10.38 ± 2.45	12.92 ± 4.29	0.192
CFTR mutation (n)	0	29	—

### Cystic fibrosis transmembrane conductance regulator and *ADGRG2* variants in congenital absence of the vas deferens patients

Twenty-four variants were identified in 50 CAVD patients after gene mutation screening; 23 patients (23/50, 46%) had *CFTR* homozygous or compound heterozygous mutations including the 5T allele [a variant of a polythymidine (Tn) polymorphism in intron 9 (NM_000493.3: c.1210-12T (5_9))], six patients (6/50, 12%) had one *CFTR* mutation, suggesting that 58% (29/50) CAVD patients had at least one *CFTR* mutation. The 5T allele was the most common variant in our CAVD populations, and the frequency of the 5T allele was 27% (27/100). A novel variant c.1767-2A>C, predicted to have an effect on the splice site, was identified in a CBAVD patient (Supplementary Table S1). No *ADGRG2* variants were identified in our CAVD patients. It is remarkable that neither *CFTR* nor *ADGRG2* variants were identified in hypospadias/CAVD patients.

### Genetic analysis in congenital absence of the vas deferens patients with hypospadias by whole-exome sequencing

WES was used on samples from CAVD patients with hypospadias to uncover the potential genetic causes. No homozygous or compound heterozygous mutations related to disorders of sex development (DSDs) or male infertility were identified after filtering by bioinformatics analysis. However, nine heterozygous or hemizygous mutations were identified as potential pathogenic genes (Table 2).

**TABLE 2:** Mutation of genes identified in hypospadias CAVD patients.

Gene (patient)	Mutation	Zygosity	Function	Allele frequency			Function prediction		
				1,000 g-all	EXAC-all	gnomAD-all	SIFT^a^	PolyPhen-2^b^	MutationTaster^c^
SEMA3E (P12)	c.580G > C	Heterozygous	Missense	0.000199681	0.0000248	0.00001629	D	D	D
NM_001178129	p.E194Q								
INVS (P12)	c.1534G > C	Heterozygous	Missense	—	—	0.00001638	D	B	N
NM_001318382	p.A512P								
CBL (P12)	c.2322T > G	Heterozygous	Nonsense	—	—	—	—	—	A
NM_005188	p.Y774*								
FRAS1(P15)	c.6401A > G	Heterozygous	Missense	—	0.00002486	0.00001625	—	B	N
NM_025074	p.N2134S								
WNT4 (P22)	c.527G > C	Heterozygous	Missense	—	—	—	T	B	D
NM_030761	p.S176T								
RSPO1 (P22)	c.466C > T	Heterozygous	Missense	—	—	0.00002844	D	D	D
NM_001242910	p.R156W								
MKKS (P22)	c.46C > A	Heterozygous	Missense	—	0.000008249	0.00000407	D	D	D
NM_018848	p.P16T								
AR (P22)	c.170_171insGCAGCAGCAGCA	Hemizygous	Nonframeshift	—	—	—	—	—	—
NM_000044	p.Q80_E81insQQQQ								
ATM (P31)	c. 4949A > G	Heterozygous	Missense	0.00239617	0.0005	0.0005	T	B	N
NM_000051	p.N1650S								

a: D means deleterious, T means tolerated; b: D means probably damaging, B means benign; c: A means disease-causing automatic; D means disease causing; N means polymorphism.

## Discussion

*CFTR* mutation is the most common genetic variation in CAVD populations, accounting for 60%–80% of CAVD patients (Chillon et al., 1995). In recent decades, with the advent of next-generation sequencing, several of these candidate genes such as *ADGRG2* (Patat et al., 2016) and copy number variants (CNVs) in *SLC9A3* (Wang et al., 2017) have been validated in some CAVD patients. Nevertheless, there is still no clear genetic diagnosis for approximately 20%–40% of CAVD patients. Hence, this study aimed to explore the variant spectrum in CAVD patients and to find new potential causative genes by WES in hypospadias/CAVD patients.

In this study, 29 out of 50 (58%) patients had at least one reportable *CFTR* variant. These results demonstrated that our CAVD population had a lower percentage than previous reports in which 70%–80% of CBAVD patients carried at least one *CFTR* mutation (Yu et al., 2012; Wang et al., 2020), which may be due to our small sample size. The variant c.1521_1523delCTT (F508del) is one of the most common variants in northern European CAVD populations, accounting for up to one-third of CAVD patients (Barratt et al., 2017; Bieth et al., 2021), whereas only two (2/50) are carriers in our CAVD patients. The 5T allele has a deleterious effect on the splicing of exon 10 and reduces the quantity of normal CFTR protein. The 5T allele polymorphism varies considerably by geographic location and ethnicity, particularly among non-Caucasian populations (Dork et al., 1997). In the current study, the frequency of the 5T allele was the most frequent variant, similar to other reports in Chinese cohorts (Du et al., 2014; Gaikwad et al., 2018; Wang et al., 2020). No *ADGRG2* variants were identified, suggesting *ADGRG2* variants may not play a major role in these CAVD populations. After Sanger sequencing, *CFTR* or *ADGRG2* mutation was not detected in 21 CAVD patients, including seven hypospadias/CAVD patients. Therefore, WES was used to further test the potential pathogenic variants in hypospadias/CAVD patients. It is to be regretted that no homozygous or compound heterozygous mutations related to male infertility were identified.

In the hypospadias/CAVD group, the patients had a relatively lower testicular volume and higher FSH level than in the non-hypospadias/CAVD group, suggesting that their fertility, especially the capacity for spermiogenesis, maybe more deficient. As is known to all, cryptorchidism is the most common congenital anomaly associated with hypospadias (Leung and Robson 2007), but CAVD with hypospadias is rarely reported. CAVD is usually associated with some congenital diseases, such as CF-related CAVD and URA-related CAVD. Particularly, numerous studies have demonstrated that URA-related CAVD is less associated with *CFTR* mutation, suggesting the etiopathogenesis that may have a different genetic background.

No homozygous or compound heterozygous mutations related to disorders of sex development (DSDs) were identified by WES. Perhaps the hypospadias/CAVD population is so small that the *CFTR* mutation rate is not accurately evaluated or the two congenital abnormalities result from an embryonic development disorder occurring in the early stage of gestation. The embryonic anomaly may involve not only the formation of urethral folds but also the derivatives of Wolffian ducts. Nevertheless, nine heterozygous or hemizygous gene mutations were selected as possible pathogenic genes, such as *SEMA3E*, *INVS*, *CBL*, *FRAS1*, and *AR*. However, the phenotypic–genotypic linkage relationship has not yet been proven between these genes and hypospadias/CAVD patients.

The loss-of-function mutations in *SEMA3E* are formally associated with CHARGE syndrome, a complex multisystem genetic disease characterized by ocular coloboma, congenital heart defects, retardation of growth, genital hypoplasia, and facial asymmetry. (Song et al., 2020), but these hypospadias/CAVD patients had no other systemic abnormalities apart from genital hypoplasia. Mutation in *INVS* is usually involved in infantile nephronophthisis, but whether it leads to infertility has not yet been reported (Otto et al., 2003). A nonsense heterozygous mutation in *CBL* was identified in a patient. *CBL* which encodes an E3-ubiquitin ligase acts as a tumor suppressor in myeloid malignancies; also, its mutation is related to a Noonan syndrome-like disorder (Tartaglia et al., 2011). Noonan syndrome is a relatively common developmental disorder with a multisystem phenotype; however, our patients only have a genitourinary phenotype. The *FRAS1* mutation is associated with Fraser syndrome. Although this syndrome presents genital anomalies, unilateral or bilateral cryptophthalmos is a typical clinical manifestation (Bouaoud et al., 2020). *RSPO1* is one of the most important genes controlling female gonadal differentiation. Previous research has shown that *RSPO1* mutation causes 46, XX testicular disorder of sex development, and that is contrary to relatively normal spermatogenesis in CAVD patients (Tallapaka et al., 2018). Mutations in the *MKKS* gene have also been shown to cause Bardet–Biedl syndrome which is characterized by pigmentary retinopathy, polydactyly, and renal abnormalities. Additionally, the *MKKS* null mouse model also failed to form spermatozoa flagella, which has no clear relevance in hypospadias or CAVD (Fath et al., 2005). A pathogenic variant in *AR* can cause androgen-insensitivity syndrome (AIS), which has undermasculinization of the external genitalia and impaired spermatogenesis, while hypospadias/CAVD patients have normal spermatogenesis. In the early stage of fetus development, testosterone promotes the formation of the internal reproductive structures (epididymis, vas deferens, prostate, and ejaculatory duct) from the Wolffian ducts, while dihydrotestosterone (DHT) induces the development of the external genitalia (Sultan et al., 2001). The level of testosterone in AIS is normal or increased, but it is normal or slightly lower in hypospadias CAVD patients. *ATM* encodes the ATM protein which activates cell-cycle checkpoints to repair the damaged DNA and prevent the presence of persistent deleterious lesions. In addition, *ATM* mutations may also be implicated in male infertility due to defective spermatogenesis (Li et al., 2013). Thus, the relevance of *ATM* to hypospadias CAVD needs to be verified more thoroughly.

Hypospadias is a common congenital condition that has been reported in over 200 syndromes, but only 30% of hypospadias cases have a clear genetic cause (van der Horst and de Wall 2017). Interestingly, few literature studies listed hypospadias in association with CAVD. All of the hypospadias/CAVD patients involved in our study were relatively healthy except for their infertility. Among the hypospadias/CAVD patients, the potential causative genes were not identified by WES in these patients, suggesting that further research needs to be carried out to elucidate the genetic background. Environmental factors, alteration of hormone levels, and epigenetic changes may play a role in the occurrence of hypospadias/CAVD.

## Conclusion

In summary, *CFTR* mutations were screened in 50 CAVD patients to make a definitive genetic diagnosis and to evaluate their genetic risk. The 5T allele is the most common variant in our CAVD populations. In hypospadias/CAVD patients, neither *CFTR* mutations nor other homozygous or compound heterozygous variants related to disorders of sex development (DSDs) or male infertility were identified by WES, but it is necessary to carry out a more detailed exploration to determine the possible pathogenesis in hypospadias/CAVD.

## Data Availability

The data that support the findings of this study have been deposited into CNGB Sequence Archive (CNSA) of China National GeneBank DataBase (CNGBdb) with accession number CNP0003603.

## Supplementary material

SUPPLEMENTARY TABLE S1 CFTR mutations identified in CAVD patients.
