# Supplementary material for: Congenital absence of the vas deferens with hypospadias or without hypospadias: Phenotypic findings and genetic considerations
Source: Front Genet. 2022 Nov 9;13:1035468. doi: 10.3389/fgene.2022.1035468 (PMC9682239; doi:10.3389/fgene.2022.1035468)
Supplement: Supplementary file 1 [file Table1.docx]

Supplementary Table S1 CFTR mutations identified in CAVD patients.

| Patients | cDNA alteration | Protein change | Mutation type | Known/  Novel | 1000g-all | EXAC-all | gnomAD-all | SIFT^a^ | PolyPhen-2^b^ | MutationTaster^c^ |
| --- | --- | --- | --- | --- | --- | --- | --- | --- | --- | --- |
| P1 | c.374T>C | p.Ile125Thr | Missense | K | 0.00159744 | 0.0006 | 0.0007 | D | B | D |
| P2 | c.2562T>G | p.Thr854Thr | Synonymy | K | 0.479233 | 0.3889 | 0.3826 | - | - | - |
|  | c.3062C>T | p.Pro1021Leu | Missense | K | - | - | - | D | D | D |
| P3 | c.1069G>A | p.Ala357Thr | Missense | K | - | - | - | D | P | D |
|  | c.2684G>A | p.Ser895Asn | Missense | K | 0.000599042 | 0.0003 | 0.0003 | T | B | N |
|  | 5T | - | Splicing | K | - | - | - | - | - | - |
| P4 | c.1070C>T | p.A357Val | Missense | K | - | - | - | T | P | D |
|  | 5T | - | Splicing | K | - | - | - | - | - | - |
| P5 | 5T | - | Splicing | K | - | - | - | - | - | - |
|  | ex.1del | - | - | K | - | - | - | - | - | - |
| P6 | 5T | - | Splicing | K | - | - | - | - | - | - |
| P7 | c.1351G>A | p.Gly451Arg | Missense | K | - | - | 8.354e-06 | D | P | D |
|  | c.3406G>A | p.Ala1136Thr | Missense | K | - | 6.598e-05 | 4.066e-05 | D | D | D |
| P8 | c.3946T>C | p. Trp1316Arg | Missense | K | - | - | - | D | D | D |
|  | c.1351G>A | p. Gly451Arg | Missense | K |  | - | 8.354e-06 | D | P | D |
| P9 | c.592G>C | p.Ala198Pro | Missense | K | - | - | 4.062e-06 | D | D | D |
|  | 5T | - | Splicing | K |  |  |  |  |  |  |
| P10 | c.650A>G | p.Glu217Gly | Missense | K | 0.00299521 | 0.0039 | 0.0047 | D | B | D |
|  | c.2909G>A | p.Gly970Asp | Missense | K | - | 8.263e-06 | 1.221e-05 | D | D | D |
| P11 | c.869+5G>A | - | Splicing | K | - | - | - | - | - | - |
|  | 5T | - | Splicing | K | - | - | - | - | - | - |
| P17 | 5T(homozygous) | - | Splicing | K | - | - | - | - | - | - |
| P18 | c.1521-1523, delTCT | Phe508del | Nonframeshift | K | - | - | - | - | - | - |
|  | c.935-937, delCTT |  | Nonframeshift | K | - | - | - | - | - | - |
| P21 | 5T | - | Splicing | K | - | - | - | - | - | - |
| P25 | 5T(homozygous) | - | Splicing | K | - | - | - | - | - | - |
| P26 | 5T(homozygous) | - | Splicing | K | - | - | - | - | - | - |
| P27 | c.4056G>C | pGln1352His | Missense | K | 0.00419329 | 0.0010 | 0.0010 | D | D | D |
|  | c.1767-2A>C |  | Splicing | N | - | - | - | - | - | - |
| P28 | c.2909G>A | p.Gly970Asp | Missense | K | - | 8.263e-06 | 1.221e-05 | D | D | D |
|  | 5T | - | Splicing | K | - | - | - | - | - | - |
| P30 | 5T | - | Splicing | K | - | - | - | - | - | - |
|  | c.1521-1523, delTCT | Phe508del | Nonframeshift | K | - | - | - | - | - | - |
| P32 | 5T(homozygous) | - | Splicing | K | - | - | - | - | - | - |
| P33 | 5T(homozygous) | - | Splicing | K | - | - | - | - | - | - |
| P35 | 5T(homozygous) | - | Splicing | K | - | - | - | - | - | - |
| P38 | 5T(homozygous) | - | Splicing | K | - | - | - | - | - | - |
| P40 | c.2812G>A | p.Val938Met | Missense | K | - | - | 4.062e-06 | D | D | D |
| P42 | c.1810A>C | p.Thr604Pro | Missense | K | - | - | - | D | D | D |
|  | 5T |  | Splicing | K | - | - | - | - | - | - |
| P45 | c.91C>T | p.Arg31Cys | Missense | K | 0.00139776 | 0.0017 | 0.0017 | D | D | D |
| P46 | c.2797A>G | p.Arg933Gly | Missense | K | - | - | - | D | D | D |
| P48 | 5T(homozygous) | - | Splicing | K | - | - | - | - | - | - |
| P50 | c.1405A>G | p.Met469Val | Missense | K | - | 1.651e-05 | 1.219e-05 | T | D | D |
|  | 5T |  | Splicing | K | - | - | - | - | - | - |

a: D means deleterious, T means tolerated; b: D means probably damaging, P means possibly damaging, B means benign; c: D means disease causing; N means polymorphism.
